# Does Education Affect Rural Women’s Trust? Evidence From China

**DOI:** 10.3389/fpsyg.2022.845110

**Published:** 2022-03-14

**Authors:** Siyu Xu, Yeye Zhao, Noshaba Aziz, Jun He

**Affiliations:** College of Economics and Management, Nanjing Agricultural University, Nanjing, China

**Keywords:** rural women, education, trust, regression discontinuity design (RDD), China

## Abstract

Trust is of great significance to the economic and social development of a country. In the case of China, the trust of rural women has undergone tremendous changes along with the development of rural areas. It is seen that the trust of rural women has changed from localized to generalized trust, and it is stated that the major factor leading to this transformation is education. To explore the phenomenon empirically, the current study uses the survey data of rural women sourced from China Family Panel Studies (CFPS) over the year 2018. Through the ordered probit model, the study reveals that education plays a significant role in influencing rural women’s generalized trust and localized trust. Through mediation analysis, the study further reveals that reliance on Internet information, access to public resources, and income are the factors mediating the relationship between education and generalized trust. Besides, the outcomes further unveil that the impact of education on localized trust is stronger when the level of mobility is low. For robustness check, the current study additionally employs a regression discontinuity model. The overall findings elucidate that education is the major factor triggering the trust of rural women in China. The findings propose that policymakers in China should imply education-oriented strategies as individuals with higher levels of education are more inclined to trust others.

## Introduction

Trust is an important social capital (Moscona et al., 2017) and is regarded as an important indicator of economic development. It is believed that mutual trust can solve common problems and compensate for both market and government failures. For instance, a high level of confidence allows economic agents to solve the problem of contract incompleteness; and further strong social ties can reduce the costs of free riding in public good provision. Trust has positive repercussions both at the macro as well as micro level. At the macro level, trust can promote economic growth, bring economic prosperity, and enhance overall social welfare in the region (Beugelsdijk and Schaik, 2004; Bjørnskov, 2012) while at the micro level, it can augment the individuals’ subjective well-being, life satisfaction, and health (Kuroki, 2011). According to the reports of the Chinese Academy of Social Sciences (CAAS), it is stated that less than half of the urban residents believe that most people in the society are trustworthy, while only 20–30% of residents trust anonymous people (CAAS, 2012, 2013, 2018). According to the database of the China Family Panel Studies (CFPS), over the years 2012, 2014, 2016, and 2018, it is found that the average trust of residents during the four samples years remained at 2.185, 1.965, 2.053, and 2.258, respectively, which reflects the overall low level of trust of people in China.

In the context of women, women’s trust cannot be ignored because it plays a significant role in the development of rural China. In the literature, it is found that the migrants’ working experience in urban areas lead to boost the living standard in their hometowns, especially in rural areas, as in the modern era, men not only permit their wives but also daughters to actively participate in economic spheres of life (Inkeles, 1975). But in the context of China, the restriction of the urban and rural household registration system could not let migrant workers to enjoy the amenities of cities. Moreover, the high living and education cost of cities restricted them from shifting their family members to cities, which subsequently lead to less welfare. Moreover, the gender division of labor and the consensus of gender, that is, males are stronger and females are feeble restrict women to confine within the domestic sphere and not let them to go out for work. With time, more rural men migrated to cities, which let rural women to incline more in agriculture farming and to develop the local industries in rural areas. As a consequence, rural women not only participated in the decision-making of household economic activities, such as borrowing and renting land, but also managed grassroot level public affairs, which led them to build trust and solve the hurdles faced by them.

In rural China, it is generally believed that the most basic way to build trust among people is by creating intimacy or close acquaintanceship. Because when the individuals have close acquaintanceship with others, the more they can trust others and more easily they can share knowledge, ethics, and other related facets. In the traditional rural society, the basic acquaintance network remained significant trust-based on kinship is found significant for rural women. But due to the migration of rural labor and the dissemination of digital technology such as the Internet in China, women’s trust has changed from blood-based relationships to society and community-based relationships. According to the social capital theory, there is difference between trust found for the general public and the trust found for intimate people (Sturgis and Smith, 2010). Trusting the general public remained a hot topic across various disciplines and cross-national researchers. Generalized trust mostly followed the standard question, such as, is it fair to trust the most people or need to remain cautious dealing with them? However, the radius of “most people” can vary across countries as it is found narrower in Confucian countries and wider in wealthy countries (Delhey et al., 2011). A myriad of studies focused on generalized trust because it helps the public to reduce transaction costs in modern society, but the researchers and academicians have also directed their interest toward exploring localized trust. Rural China is regarded as a society of acquaintances or a society of human relations, and localized trust is highly prevalent and acknowledged as a social capital. For instance, people rely on localized trust while borrowing money from their relatives and neighbors or renting land, and they do not need formal contract or agreement. The higher the localized trust, the easier it is to live in the countryside that lead to them many other significant outcomes, such as local funding decisions and non-profit organizations (Leigh, 2006; Tu and Bulte, 2010).

Based on the above discussion, the current study explores the trust phenomenon from two perspectives encompassing both generalized trust and localized trust. It is generally believed that men and women are different based on their interdependency such as women are more relationally interdependent, while men are more collectively interdependent (Gabriel and Gardner, 1999; Maddux and Brewer, 2005). In social activities, trust and participation are positively correlated, even though the results on participation and trust are diverse. The more women participate in social activities, the more they can interact with people and can build the trust in them (Alesina and Ferrara, 2002). Even the participation of women in rural communities in developing countries empowers them and enables them to achieve many other objective repercussions such as food security and health (Aziz et al., 2020a,b, 2021a,b; Wei et al., 2021). In developing countries, trust is also seemed as vital, as formal markets seemed underdeveloped, so in the informal circumstances, reputation significantly matters (Tu and Bulte, 2010; Asadullah, 2017). Li et al. (2017) and Chen et al. (2020) also stated that regions where formal institutions are weak, trust plays a prominent role. Back in the 1980s and 1990s, it is found that top-down market reform and liberalization in many transitions and developing countries failed to yield the desired results due to the absence of sufficient “trust” at the beginning (Easterly, 2005). In low-trust societies, people were more likely to use informal arrangements, they usually trade with people whom they know (Tu and Bulte, 2010). But in modern society where transaction anonymity is becoming more assertive, trust plays an important role in maintaining cooperation between people and promoting economic growth and development (Zak and Knack, 2001). It has been proven by many studies that education plays an important role in boosting the trust of people. Helliwell and Putnam (1999) in their studies proved that an increase in average education increases trust. But how effectively the education improves the trust of rural women is an important issue that needs to be addressed and resolved, especially in the context of China where gender inequality remained rising in the years back in the 1980s and 1990s.

In the prevailing literature, bulk of studies explored the gender inequality in rural China, especially in the context of education. In rural areas, the conservative families still prefer boys over girls, as they believe that they will get more support from boys in their old age (Hannum et al., 2009). Moreover, rural women usually get married at a younger age. Furthermore, in 2003, girls dropped out more than boys and when wage started raising in the later years, the boys also started to quit education (Liu and Rozelle, 2020). By the year 1990, the gender disparities remained concentrated in rural areas of China as children with more siblings competed for educational resources. The costs of education have proven to be a burden for poor families (Connelly and Zheng, 2007). So all these facets led to the prevailing of gender inequality, especially in the context of education, throughout the 1980s and 1990s in rural areas of China and inequality led women to trust less (Jordahl, 2009). In many studies, it is stated that the trust of people on each other within a country is also linked with various other factors, such as governance, bureaucratic corruption, and level of schooling attained (Asadullah, 2017). Now the education in rural areas in China has made tremendous changes, as in the early 1960s, one-third of the Chinese were illiterate; now, only fewer than 5 percent are illiterate (Peng, 2011). Interestingly, in 1986, China’s central government passed a law requiring 9 years of education, and over the subsequent several years, provincial authorities began enforcing compulsory education in their localities (Fang et al., 2012). In China, rural households generally believe that women’s educational returns are lower than men’s, so these women are forced to drop out of school to work or get married. But later, the “Compulsory Education Law of the People’s Republic of China” enforced that school-age children must be educated, so rural school-age women also got chances to get education. As a consequence, the number of children attending school in rural areas increased to more than 90% by the year 2014 as compared to 1980 (Yue et al., 2018). It is found that education can improve people’s basic cognition level including trust. So based on this argument it is believed that education can improve rural womens’ relationship with the society and boost their trust level. Trust is important for the smooth operation of the society. Now the question arises, why women with increased education still have low trust levels in rural China? To answer this question the current study explores the factors influencing the trust of rural women.

The current study contributes to the literature by the following ways such as the study first uses the CFPS data for the year 2018 to explore the relationship between education and rural women’s trust, both localized and generalized. Second, the study explores the mechanisms through which education influences trust. To explore the mechanism, the current study employs reliance on Internet information, access to public resources, and income as mediators. Third, it is believed that trust of rural women has changed from localized to generalized trust but it varies along different space and time. So the current study applies the heterogeneity test to unravel the heterogeneity among the sampled data. Fourth, due to the “Compulsory Education Law of the People’s Republic of China” implemented in China, a lot of women were allowed to receive education. Considering whether this part of the additional education years has an impact on the trust of rural women, the current study additionally employs the regression discontinuity model to explore the causal relationship between education and trust.

The rest of the article is organized as follows. The following section presents the literature review and conceptual framework with hypothesis. Section III is a methodology, including a description of the data and model used in this article. Section IV details the results of our analysis. Section V discusses the findings and the final section, Section VI, concludes the study with proper policy implication and also offers some directions for future research.

## Literature Review

There is a large literature concerning the effect of education on trust. Therefore, this section is an attempt to unveil the previous efforts of the researchers. Some studies discuss trust toward specific social groups by using different methodologies. For instance, Borgonovi (2012) used multilevel random intercept models to systematically test for country-level differences in the association between education and levels of interpersonal trust toward migrants in a sample of 21 European countries. The results suggested that better educated individuals trust migrants more than poorly educated individuals. Another study of Leigh (2006) used precise data on neighborhood characteristics and found that an extra year of education is associated with increased localized trust. Other studies also discussed the same phenomenon and exhibited that various factors may increase general trust. Sønderskov and Dinesen (2014) in their study revealed that promoting higher education can foster social trust in Denmark over the last 30 years. Alesina and Ferrara (2002) used individual-level data from the United States (US) localities and found that individual characteristics, such as education and income, and the community characteristics influence the peoples’ trust. Though various studies revealed the positive effect of education on trust, however, Oskarsson et al. (2017) on the other side found contradictory findings. They used a within-twin-pair approach and found an insignificant relationship between education and social trust.

In the context of women, many studies used different factors to explore the relationship with trust. In this vein, Maddux and Brewer (2005) designed an online trust-dilemma game and revealed that women preferred to trust those individuals with whom they have direct or indirect interaction relationships, while men trust only those individuals with whom they share group memberships. In another study, Fisman and Khanna (1999) explored the trust from the perspectives of digital technology such as Internet access and found that public facilities such as telephones affect social trust, as these help people to exchange information and also help residents to understand each other’s preferences. Besides, it boosts the collective identity among residents, thereby enhancing the level of trust among them. In another study, Steinhardt (2012) used the comparative leverage provided by a political and sociocultural variation to investigate the plausible reasons for the high levels of trust in Mainland China and found that a comparatively strong link exist between institutional confidence and trust. The study suggested that high confidence in institutions significantly contributes to the high levels of generalized trust. Based on Steinhardt’s study, Tao et al. (2014) used an instrumental variable approach and untangled the relationship between social and political trust in contemporary China. The findings revealed that political trust enhances the social trust. Likewise, Asadullah (2017) in the case of Bangladesh also perceived that institutional trust positively influences the interpersonal trust. In China, though the Chinese government has increased the public resources, it is far from the needs of the people. Local people undoubtedly compete for public resources such as education and medical care that unfavorably influences the trust of people on government institutions. Thus, a strong relationship exist between public resource access and the government institutions. Moreover, it is also believed that mobility is another factor affecting trust. Alesina and Ferrara (2002) in their study pointed out that an individual who has not been living in the current place of residence for a long time may be less intimate with others and less inclined to trust others, because he or she may not know other people enough. Likewise, if people live in a community in which everyone is “transitory” (not permanently residing in the area), it may also reduce the interaction of residents and ultimately trust. But with the increase of an individual’s education and income, trust can also increase (Alesina and Ferrara, 2002). Jordahl (2009) also found that income influences trust.

Based on the above literature, it is apparent that several studies have proven a significant positive effect of education on trust (Leigh, 2006; Borgonovi, 2012; Sønderskov and Dinesen, 2014), but these studies ignored the mechanism through which education influences trust. So, moving ahead and filling this gap in the literature, the current study explores the mechanisms by which education affects trust especially in the context of rural Chinese women. Moreover, unlike the previous studies, the current study uses the joint aspects of trust, that is, generalized trust and local trust. The joint aspects will better yield the findings.

### Conceptual Framework and Hypotheses Development

There is a widespread research on trust. By following the study of Arrow (1972) and Fukuyama (1995) who discussed the relationship between trust and economic success, economists, try to identify who trust and measure trust by using survey questionnaires (Glaeser et al., 2000; Alesina and Ferrara, 2002; Asadullah, 2017; Moscona et al., 2017). The prevailing literature acknowledges that education positively affects trust (Alesina and Ferrara, 2002; Leigh, 2006; Borgonovi, 2012; Sønderskov and Dinesen, 2014). While discussing generalized trust, it is argued that besides education, trust can also be affected by several other factors such as reliance on Internet information, access to public resources, and income from rigorous academic studies (Fisman and Khanna, 1999; Alesina and Ferrara, 2002; Maddux and Brewer, 2005). Meanwhile, it is also debated that education has a crucial association with several other factors such as reliance on Internet information, access to public resources, and income (Atkinson et al., 2010; Sǎseanu and Petrescu, 2012; Yang and Gao, 2018). It reflects the point that educated individuals better understand the positive impacts of associational activities and collective action on society than those with less formal education (Rupasingha et al., 2006). Internet is an open platform where anyone can publish information on it. Concerning the reliance on Internet information, the more information people exchanged through Internet, the more generalized trust they gained. On the other side, individuals’ education is likely to lead toward inequality in income level, family economic status, or social status. Such disparities are expected to influence individuals’ access to public goods such as education and medical care for the next generation. In this way, the inequality of access to public goods caused by education may reduce trust.

**FIGURE 1:**
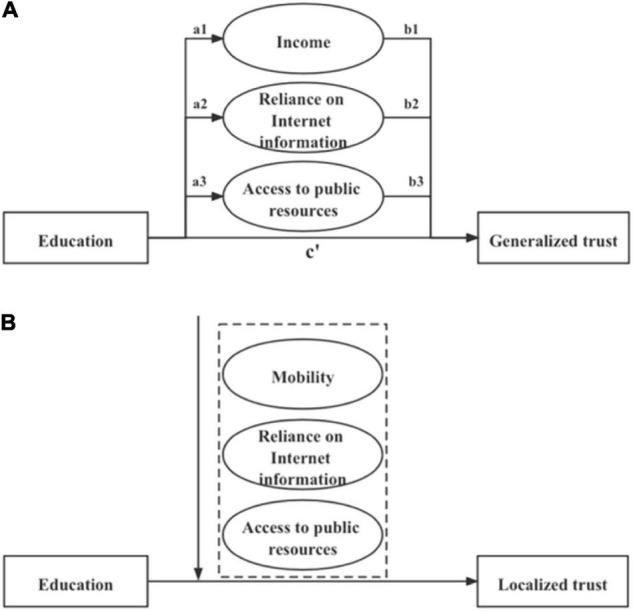
**(A)** Framework of mediating effect of generalized trust. **(B)** The framework of moderating effect of localized trust.

So based on the above discussion, this study formulates the following hypotheses for generalized trust (see Figure 1A):

H1: Education has a positive effect on rural women’s generalized trust.

H2: Reliance on Internet information, access to public resources, and income mediate the relationship between education and generalized trust, that is, the changes in rural women’s reliance on Internet information, access to public resources, and income brought by education contribute to the increased level of generalized trust.

As far as localized trust is concerned, education may have a different effect. In the case of Chinese rural society, traditional culture is an important factor when discussing localized trust, especially, “the pattern of difference sequence.” This pattern includes not only blood kinship but also geographic relationships. Therefore, in rural China where traditional associations dominate, rural women’s localized trust is related to the social structure of “the pattern of difference sequence,” that is, trust based on the long-term interaction of blood and neighbor relationships. With the advancement and progression of Internet, rural women are believed to rely more on Internet information source as compared to traditional mode of gaining information. Moreover, when rural women realize that access to public resources is obtained from open sources rather than from acquaintances that are often prevalent in rural areas, their traditional trust is also likely to change. So it is hypothesized that reliance on Internet information and access to public resources significantly influence rural women’s localized trust.

Further, in China, education provides mobility opportunities to rural women (Crown et al., 2021), and hukou affects labor market inequality between rural migrants and urbanite workers (Xiao and Bian, 2018). Away from the original living environment, mobility may influence individuals’ previous views on people and things; therefore, individual migration naturally influence their localized trust. Mobility affects localized trust, because most migrants are inclined to communicate with people, especially with whom they are familiar. So the current study hypothesizes that the impact of education on localized trust is strong when the level of reliance on Internet information, access to public resources, and mobility is low. So the reliance on Internet information, access to public resources, and mobility are hypothesized to have moderating effects between education and localized trust. Therefore, the study formulates the following hypotheses (see Figure 1B):

H3: Education has a significant positive effect on rural women’s localized trust.

H4: Reliance on Internet information, access to public resources, and mobility have moderating effects between education and localized trust.

## Methodology

### Data Sources and Participants

The data used in this article are drawn from the CFPS for the year 2018. It is a national-wide comprehensive and longitudinal social survey that includes data from 31 provinces of China and encompasses a large variety of social phenomena regarding contemporary China. Extensive information is collected through computer-assisted person-to-person interviews of all family members. The age of the sample is from 16 to 80 years old. The questionnaires not only cover a wide range of topics about Chinese society, economy, population, education, and health by tracking three levels of individuals, families, and communities, but also consist of integraded modules for rural and urban areas, such as information about family structure and family members, migrant mobility, event history (e.g., history of marriage, education, and employment), cognitive ability, and child development (Xie and Hu, 2014). In this study, we screened the women whose hukou was in rural areas at the age of 12, and the total final sample size found was about 5,215.

### Study Variables

Many studies adapted the trust question used in the World Values Survey, that is, “Generally speaking, would you say that most people can be trusted or that you need to be very careful when dealing with people?” The average response to the generalized trust question is the standard measure of social capital or trust in the empirical cross-country literature (Zak and Knack, 2001; Bjørnskov, 2012). However, some studies are concerned about the exact content of this measure of trust (Asadullah, 2017). A high level of trust produces a large amount of general trust, given that the radius of trust is wide (Delhey et al., 2011). Thus, to distinguish between localized trust and generalized trust, this article divides trust into two parts, trust in neighbors and trust in strangers. In the current study, localized trust and generalized trust are dependent variables, which are based on answers to the question “How much trust can be scored by neighbor” and “How much trust can be scored by stranger.” Those are on a scale ranging from 1 (don’t trust at all) to 10 (trust completely). The importance of the Internet is a proxy for the reliance on Internet information, and has index ranges from 1 (no importance at all) to 5 (complete importance). Evaluation of own county/city government is a proxy for the access to public resources, on a scale of 1–5 (where 1 = low; 5 = high). Hukou is changed is a proxy for mobility (1 = hukou is changed; 0 = hukou is not changed). The data also includes other variables that reflect personal characteristics, including age, marriage, religious group, confidence in the future, the harmony of the family, member of Communist Party of China (CPC), health, and income. Table 1 presents the descriptive statistics of all variables.

**TABLE 1:** Descriptive statistics.

Variable	Value	Mean	Std.	Min	Max
Education	1 = illiteracy; 2 = primary school; 3 = junior high school; 4 = senior high school; 5 = university or above	2.863	1.24	1	5
Age	Years old	36.560	11.34	16	80
Marriage	1 = married; 0 = unmarried	1.985	0.32	0	1
Religious group	1 = yes; 0 = no	0.027	0.16	0	1
Confidence in the future	1–5 means the lowest level to the highest level	4.136	0.89	1	5
Harmonious of family	1–5 means the lowest level to the highest level	4.643	0.70	1	5
CPC member	1 = yes; 0 = no	0.012	0.11	0	1
Health	1–5 means the lowest level to the highest level	2.862	1.14	1	5
Income	Yuan (log)	5.132	5.08	0	12.8
Importance of Internet	1–5 means the lowest level to the highest level	3.463	1.54	1	5
Evaluation of own county (city) government	1–5 means the lowest level to the highest level	3.365	0.92	1	5
Popularity	0–10 means the lowest level to the highest level	6.984	1.84	0	10
Change of Hukou	1 = yes; 0 = no	0.048	0.21	0	1
Trust strangers	0–10 means the lowest level to the highest level	2.102	2.12	0	10
Trust neighbors	0–10 means the lowest level to the highest level	6.486	2.05	0	10

## Empirical Approach

### Generalized Trust

This study applies the following regression equation to test H1 and H2. The value of generalized trust is discrete and orderly and in this case, ordinary least squares (OLS) estimation may lead to biased results. So ordered probit model is used for regression analysis as it is proven that probit model can estimate the non-linear effects more accurately. The ordered probit model is structured as follows:


$$T\,r\,u\,s\,t_{i\,g}^{*}=\beta_{0}+\beta_{1}\,E\,d\,u+\delta \,I\,n\,c\,o\,m\,e+\gamma \,\mathrm{Reliance}\,\mathrm{on}\,\mathrm{Internet}\text{information}+\sigma \,\mathrm{Access}\,\mathrm{to}\,\mathrm{public}\,\mathrm{resources}+\mu_{g}$$

(1) T⁢r⁢u⁢s⁢ti⁢g⁢{1i⁢f⁢T⁢r⁢u⁢s⁢ti⁢g*≤α1 2i⁢f⁢α1<T⁢r⁢u⁢s⁢ti⁢g*≤α2 3i⁢f⁢α2<T⁢r⁢u⁢s⁢ti⁢g*≤α3 4i⁢f⁢áá3<T⁢r⁢u⁢s⁢ti⁢g*≤α4 5i⁢f⁢α4<T⁢r⁢u⁢s⁢ti⁢g*≤α5 6i⁢f⁢α5<T⁢r⁢u⁢s⁢ti⁢g*≤α6 7i⁢f⁢α6<T⁢r⁢u⁢s⁢ti⁢g*≤α7 8i⁢f⁢α7<T⁢r⁢u⁢s⁢ti⁢g*≤α8 9i⁢f⁢α8<T⁢r⁢u⁢s⁢ti⁢g*≤α9 10i⁢f⁢α9<T⁢r⁢u⁢s⁢ti⁢g*≤α10

(2) $$T\,r\,u\,s\,t_{i\,g}^{*}=c\,E\,d\,u+e_{1}$$

(3) $$X=a_{i}\,E\,d\,u+e_{2}$$

(4) $$T\,r\,u\,s\,t_{i\,g}^{*}=c^{\prime }\,E\,d\,u+b_{i}\,X+e_{3}$$

where $T\,r\,u\,s\,t_{i\,g}^{*}$ is the unobservable generalized trust and i represents individual. Consistent with H1, we expect a significant positive coefficient on β_1_.

Between the formula (2) and (4), X presents mediators, such as reliance on Internet information, access to public resources, and income while *e*_1_,*e*_2_,*e*_3_are the error terms at random. The coefficient c of the formula (4) is the total effect of education on generalized trust. The coefficient *a_i_* in the formula (5) is the effect of mediators and the coefficient *b_i_* in the formula (6) is the effect of the mediators on the generalized trust after controlling the effect of education. The coefficient c’ is the direct effect of education on the generalized trust after controlling the effect of the mediators. The mediating effect is equal to the product of the coefficient *a_i_* and coefficient *b_i_*, which is recorded as *a_i_b_i_*. The steps of mediating effect analysis are as follows: first, test whether the total effect C of education on generalized trust is significant or not. If it is not significant, it means that the effect of education on generalized trust is not significant, and there is no need to test the mediating effect. If that is significant, then proceed to the next step. Second, test the significance of the coefficient *a_i_* and *b_i_*. If both of them are significant, it indicates that the indirect effect is significant and then moves to the fourth step to continue the test. If at least one of them is not significant, then continue the third step test. The bootstrap method is used to test whether the indirect effect is significant. If the indirect effect is not significant, it indicates that there is no mediating effect. The fourth step is to compare the symbols of *a_i_b_i_* and c’. If the symbols are the same, there is a mediating effect, and the proportion of mediating effect in the total effect is reported as *a_i_b_i_*/c.

### Localized Trust

The value of localized trust is discrete and orderly, so the same procedure is followed for localized trust as discussed above for generalized trust. The equation is structured subsequently as follows:


$$T\,r\,u\,s\,t_{i\,l}^{*}=\alpha_{0}+\alpha_{1}\,E\,d\,u+\varphi \,\mathrm{Reliance}\,\mathrm{on}\,\mathrm{Internet}\text{information}+\eta \,\mathrm{Access}\,\mathrm{to}\,\mathrm{public}\,\mathrm{resources}+\theta \,M\,o\,b\,i\,l\,i\,i\,t\,y$$

$$+\lambda \,E\,d\,u*\mathrm{Reliance}\,\mathrm{on}\,\mathrm{Internet}\,\mathrm{information}+\kappa \,E\,d\,u*\text{Access}\mathrm{to}\,\mathrm{public}\,\mathrm{resources}+\omega \,E\,d\,u*M\,o\,b\,i\,l\,i\,t\,y+\mu_{l}$$

(5) $$T\,r\,u\,s\,t_{i\,l}\,\left\{ \begin{array}{cc} 1\,i\,f\,T\,r\,u\,s\,t_{i\,l}^{*}\leq \alpha_{1} & \\ 2\,i\,f\,\alpha_{1}<T\,r\,u\,s\,t_{i\,l}^{*}\leq \alpha_{2} & \\ 3\,i\,f\,\alpha_{2}<T\,r\,u\,s\,t_{i\,l}^{*}\leq \alpha_{3} & \\ 4\,i\,f\,\alpha_{3}<T\,r\,u\,s\,t_{i\,l}^{*}\leq \alpha_{4} & \\ 5\,i\,f\,\alpha_{4}<T\,r\,u\,s\,t_{i\,l}^{*}\leq \alpha_{5} & \\ 6\,i\,f\,\alpha_{5}<T\,r\,u\,s\,t_{i\,l}^{*}\leq \alpha_{6} & \\ 7\,i\,f\,\alpha_{6}<T\,r\,u\,s\,t_{i\,l}^{*}\leq \alpha_{7} & \\ 8\,i\,f\,\alpha_{7}<T\,r\,u\,s\,t_{i\,l}^{*}\leq \alpha_{8} & \\ 9\,i\,f\,\alpha_{8}<T\,r\,u\,s\,t_{i\,l}^{*}\leq \alpha_{9} & \\ 10\,i\,f\,\alpha_{9}<T\,r\,u\,s\,t_{i\,l}^{*}\leq \alpha_{10} & \end{array}\right.\,\left(5\right)$$

where $T\,r\,u\,s\,t_{i\,l}^{*}$ is the unobservable localized trust, and to test H3, we expect a significant positive coefficient on β_1_. Using the interaction of education with reliance on Internet information, access to public resources, and mobility, that is, education*reliance on Internet information, education*access to public resources, and education *mobility, to test H4, the H4 predicts that the effect of reliance on Internet information, access to public resources, and mobility are weaker for education. Therefore, we expect a negative coefficient on education*reliance on Internet information, education*access to public resources, and education *mobility.

## Results

In the current study, responses to trust neighbors are used as dependent variables. The main individual-specific variables of interest are income, the reliance on the Internet, evaluation of own county (city) government, and change of Hukou. In the prevailing literature, these are considered as important indicators for social trust. The study also includes control variables such as individual characteristics including age, marriage, health, confidence in the future, and socioeconomic conditions of the individual including member of the CPC, popularity, harmonious family, and religious group. As already mentioned the current study explored trust from two perspectives such as localized trust and generalized trust. So the current study first presents the effects of education on rural women’s generalized trust and then localized trust, and also presents the mechanism influencing these trusts and further presents robustness check results to endorse the study findings.

### Results of the Effect of Education on Rural Women’s Generalized Trust

Table 2 shows the results of the ordered probit estimates that explored the effect of education on rural women’s generalized trust. The results show that education is statistically significant in the case of trusting strangers. The results provide preliminary support for H1, indicating that rural women with higher education levels are more likely to trust strangers. Further, the results of income, reliance on Internet information, and access to public resources are all found statistically significant entailing that education plays positive effects on trusting strangers. On the other hand, mobility is not statistically significant in this model. In other words, the effect of mobility on generalized trust is not significant. A recent study by Si et al. (2021a) in China also confirmed that the households’ poor socioeconomic conditions restrict families to secure welfare.

**TABLE 2:** Ordered probit estimates of the effect of education on rural women’s generalized trust.

Variable	(1)	(2)	(3)	(4)
	Trust strangers	Trust strangers	Trust strangers	Trust strangers
Education	0.127***	0.103***	0.097***	0.098***
	(0.016)	(0.017)	(0.017)	(0.018)
Age	−0.001	0.001	0.000	−0.000
	(0.002)	(0.002)	(0.002)	(0.002)
Marriage	−0.165***	−0.181***	−0.170***	−0.164***
	(0.050)	(0.050)	(0.050)	(0.051)
Religious group.	−0.049	−0.061	−0.080	−0.080
	(0.091)	(0.092)	(0.092)	(0.092)
Confidence in the future	−0.011	−0.014	−0.019	−0.019
	(0.018)	(0.018)	(0.018)	(0.018)
Harmonious of family	−0.059***	−0.065***	−0.069***	−0.069***
	(0.022)	(0.022)	(0.022)	(0.022)
CPC member	0.053	0.069	0.068	0.070
	(0.134)	(0.134)	(0.134)	(0.134)
Health	−0.022	−0.022	−0.018	−0.018
	(0.014)	(0.014)	(0.014)	(0.014)
Income	0.006*	0.005	0.006*	0.006*
	(0.003)	(0.003)	(0.003)	(0.003)
Reliance on Internet information		0.047***	0.046***	0.046***
		(0.013)	(0.013)	(0.013)
Access to public resources			0.096***	0.097***
			(0.016)	(0.016)
Mobility				−0.087
				(0.070)
Pseudo R2	0.0113	0.0120	0.0138	0.0139
N	5,215	5,215	5,215	5,215

Standard errors are in parentheses. ***p < 0.01 and *p < 0.1.

Further, to examine the mediating effect between education and rural women’s generalized trust, the current study used multiple mediator models (Preacher and Hayes, 2008) by taking income, reliance on Internet information, and access to public resources as mediators and employed the mediation analysis by taking the following steps (see Tables 3, 4). First, the study used the OLS model to estimate the total effect (C) of education on rural women’s generalized trust, and the coefficient is found to be 0.218, which is significant at the 1% statistical level, that reflects that mediation analysis can be effectively carried out. Second, the mediation effect *a*_*i*_*b*_*i*_of the mediators (income, reliance on Internet information, and access to public resources) on the effect of education on rural women’s generalized trust is explored. Since the estimated values of *a_i_b_i_* are all found significant and the coefficients of *a_i_b_i_* are all found positive as the coefficient of c, it reveals that the mediation effect of income, reliance on Internet information, and access to public resources exist and the mediating effect ratio (*a_i_b_i_*/c) is 11.13, 25.8, and 3.65%, respectively. This explains to some extent that about 11.13, 25.8, and 3.65% of the effect of education on rural women’s generalized trust is achieved through income, reliance on Internet information, and access to public resources. These findings support the hypothesis H2.

**TABLE 3:** Test the mediating effect of education on trusting strangers.

Variable	Coef.	Std. Err.	z	*P* > z	[95% Conf. Interval]	
**Income**						
Education	1.910	0.050	38.01	0.000	1.812	2.009
**Reliance on internet information**						
Education	0.750	0.014	54.82	0.000	0.724	0.777
**Access to public resources**						
Education	0.043	0.010	4.15	0.000	0.022	0.063
**Trust stranger**						
Income	0.0127	0.006	1.97	0.048	0.000	0.025
Reliance on Internet information	0.075	0.024	3.19	0.001	0.029	0.122
Access to public resources	0.185	0.031	5.92	0.000	0.124	0.247
Education	0.218	0.031	7.02	0.000	0.157	0.278

**TABLE 4:** Bootstrap test.

Path	Observed Coef.	Std. Err.	z	*P* > |z|	Normal-based [95% Conf. Interval]	
Education → Income → Generalized trust	0.024	0.013	1.87	0.062	−0.001	0.050
Education →Reliance on Internet information→ Generalized trust	0.057	0.018	3.20	0.001	0.022	0.091
Education →Access to public resources→ Generalized trust	0.008	0.002	3.18	0.001	0.003	0.013
Total	0.493	0.084	5.87	0.000	0.3280	0.6570

Furthermore, to test the significance and CI of the mediation analysis, the current study employed a bootstrap analysis by following the Sobel test (Wen et al., 2010). According to the test, the findings obtained in the study using the Bootstrap test for 500 iterations are found significant. This further confirms that education affects rural women’s generalized trust, and the logical chain of “education→income/reliance on Internet information/access to public resources→generalized trust” is also verified.

### Results of the Effect of Education on Rural Women’s Localized Trust

This section presents the estimates of the effect of education on rural women’s localized trust. The results in Table 5 show that there is a statistically significant and positive effect of education on trusting neighbors. These results provide support to hypothesis H3, suggesting that rural women with higher education levels are more likely to trust their neighbors. Reliance on Internet information and access to public resources also significantly influence the rural women’s localized trust. On the contrary, mobility has an expectedly negative and statistically significant effect on trusting neighbors, which means if women change their Hukou and migrate to other communities, they will be less likely to trust their neighbors. Unlike the generalized trust results, the findings of the harmonious of the family is found to be positively significant, that is, the more harmonious the family, the more they can trust relatives and friends around them, and the more localized trust they will have.

**TABLE 5:** Ordered probit estimates of the effect of education on rural women’s localized trust.

Variable	(5)	(6)	(7)	(8)
	Trust neighbors	Trust neighbors	Trust neighbors	Trust neighbors
Education	0.060***	0.037**	0.031*	0.032*
	(0.015)	(0.017)	(0.017)	(0.017)
Age	0.012***	0.014***	0.013***	0.013***
	(0.002)	(0.002)	(0.002)	(0.002)
Marriage	−0.077	−0.091*	−0.079	−0.071
	(0.049)	(0.050)	(0.050)	(0.050)
Religious group.	0.043	0.033	0.016	0.015
	(0.088)	(0.088)	(0.088)	(0.088)
Confidence in the future	0.197***	0.194***	0.190***	0.190***
	(0.017)	(0.017)	(0.017)	(0.017)
Harmonious of family	0.114***	0.109***	0.106***	0.106***
	(0.021)	(0.021)	(0.021)	(0.021)
CPC member	−0.203	−0.189	−0.191	−0.189
	(0.132)	(0.132)	(0.133)	(0.133)
Health	−0.048***	−0.048***	−0.044***	−0.045***
	(0.013)	(0.013)	(0.013)	(0.013)
Income	−0.001	−0.003	−0.002	−0.002
	(0.003)	(0.003)	(0.003)	(0.003)
Reliance on Internet information		0.044***	0.043***	0.043***
		(0.012)	(0.012)	(0.012)
Access to public resources			0.094***	0.095***
			(0.016)	(0.016)
Mobility				−0.113*
				(0.067)
Pseudo R2	0.0144	0.0150	0.0168	0.0169
N	5,215	5,215	5,215	5,215

SEs are in parentheses. ***p < 0.01, **p < 0.05, and *p < 0.1.

By following the literature, the results of moderation are reported in Table 6, which shows that there exist moderation effects between education and localized trust through reliance on Internet information, access to public resources, and mobility. Among these three variables, mobility has a negative moderating effect of education on localized trust, which means mobility weakens that effect. Rural women with higher education are more likely to change their Hukou, but when they moved to the cities, they lack a sense of identity for themselves. These rural women do not consider themselves as a member of the cities, so they reduce localized trust. Hence, H4 is not supported, because only mobility has moderating effects between education and localized trust.

**TABLE 6:** Order probit estimates of moderating effects of the effect of education on rural women’s localized trust.

Variable	(9)	(10)	(11)
	Trust neighbors	Trust neighbors	Trust neighbors
Education	0.037**	0.043	0.074*
	(0.017)	(0.037)	(0.045)
Age	0.013***	0.013***	0.013***
	(0.002)	(0.002)	(0.002)
Marriage	−0.069	−0.074	−0.072
	(0.050)	(0.050)	(0.050)
Religious group.	0.014	0.016	0.016
	(0.088)	(0.088)	(0.088)
Confidence in the future	0.191***	0.190***	0.190***
	(0.017)	(0.017)	(0.017)
Harmonious of family	0.107***	0.106***	0.106***
	(0.021)	(0.021)	(0.021)
CPC member	−0.193	−0.187	−0.184
	(0.133)	(0.133)	(0.133)
Health	−0.044***	−0.044***	−0.045***
	(0.013)	(0.013)	(0.013)
Income	−0.002	−0.002	−0.002
	(0.003)	(0.003)	(0.003)
Reliance on Internet information	0.042***	0.050**	0.043***
	(0.012)	(0.025)	(0.012)
Access to public resources	0.095***	0.095***	0.129***
	(0.016)	(0.016)	(0.037)
Mobility	0.211	−0.113*	−0.113*
	(0.195)	(0.067)	(0.067)
Education* Mobility	−0.099*		
	(0.056)		
Education*Reliance on Internet information		−0.003	
		(0.009)	
Education*Access to public resources			−0.013
			(0.012)
Pseudo R2	0.0171	0.0169	0.0170
N	5,215	5,215	5,215

SEs are in parentheses. ***p < 0.01, **p < 0.05, and *p < 0.1.

### Results of the Change of Rural Women’s Trust

The previous analysis has confirmed the effect of education on rural women’s trust. However, the above results are only the average effect of the whole sample and do not consider the group heterogeneity of rural women. So, moving forward, the current study should group the sample based on generation and region to obtain more detailed research conclusions. From the perspective of generations, according to previous literature, rural women are divided into two groups, that is, those who were born before 1980 and those who were born after 1980. The region is divided into east, middle, and west. According to the estimates presented in Table 7, it is apparent that education has a significant positive effect on the generalized trust of rural women born after 1980. However, education has no significant effect on the generalized trust of rural women born before 1980.

**TABLE 7:** Order probit estimates of the effect of education on rural women’s trust: generational differences.

	Trust strangers		Trust neighbors	
	After 1980	Before 1980	After 1980	Before 1980
Education	0.174***	−0.003	0.032	0.019
	(0.022)	(0.029)	(0.021)	(0.026)
Reliance on Internet information	0.038**	0.071***	0.056***	0.019
	(0.018)	(0.019)	(0.017)	(0.018)
Access to public resources	0.114***	0.067***	0.102***	0.089***
	(0.021)	(0.026)	(0.020)	(0.025)
Mobility	−0.175**	0.063	−0.210***	0.180
	(0.078)	(0.152)	(0.075)	(0.147)
Control Variable	Y	Y	Y	Y
Pseudo R2	0.0178	0.0082	0.0186	0.0144
N	3264	1951	3264	1951

SEs are in parentheses. ***p < 0.01 and **p < 0.05.

Moreover, there are differences in the level of economic development among provinces in different regions of China, so there are also different levels of trust in different regions. This study also divides all provinces of China into western provinces, central provinces, and eastern provinces according to their geographical locations. Table 8 shows the result of the effect of education on rural women’s trust based on regions. According to Table 8, it is shown that education has a significant effect on the rural women’s generalized trust and localized trust in the central provinces, while in the case of eastern China, the results are only found significant for rural women’s generalized trust.

**TABLE 8:** Ordered probit estimates of the effect of education on rural women’s trust: regional differences.

	Trust strangers			Trust neighbors		
	West	Middle	East	West	Middle	East
Education	0.037	0.194***	0.132***	0.024	0.092***	−0.011
	(0.028)	(0.035)	(0.029)	(0.027)	(0.034)	(0.028)
Reliance on Internet information	0.033	0.084***	0.047**	0.034*	0.039	0.056***
	(0.021)	(0.026)	(0.022)	(0.020)	(0.025)	(0.021)
Access to public resources	0.110***	0.138***	0.070***	0.072***	0.133***	0.097***
	(0.027)	(0.034)	(0.026)	(0.026)	(0.033)	(0.025)
Mobility	−0.160	0.042	−0.136	−0.130	0.015	−0.158
	(0.119)	(0.141)	(0.108)	(0.113)	(0.137)	(0.104)
Control Variable	Y	Y	Y	Y	Y	Y
Pseudo R2	0.0124	0.0249	0.0156	0.0150	0.0256	0.0177
N	1790	1299	2113	1790	1299	2113

SEs are in parentheses. ***p < 0.01, **p < 0.05, and *p < 0.1.

### Robustness Results

To check the robustness of results, this study used a regression discontinuity design (RDD) to complete robustness checks and extensions to the core analysis. The reason for conducting this phenomenon is because China, in 1986, passed the “Compulsory Education Law” for the People’s Republic of China. According to the law, the country implemented a 9-year compulsory education system and enforced that children who were at the age of six are required to complete their compulsory education. Even the children who were out-of-school under the age of 15 had to return to school to study, but no such requirement was levied for out-of-school children who were above 15 years of age. Therefore, after the compulsory education law was promulgated, a lot of women were allowed to receive education. However, this part of the additional education is brought about by policies, so it has nothing to do with innate factors. Considering whether this part of the additional education years has an impact on the trust of rural women, the current study employed the regression discontinuity model to further explore the phenomenon between education and the trust of rural women. The compulsory education is accepted at the age of 6, and Chinese primary school enrollment starts in September each year. Therefore, individuals affected by the Compulsory Education Law promulgated in 1986 are those individuals who were born after September 1971. To implement break point regression, the sample in this article only includes those individuals who were born within 10 years before and after the policy effect, that is, individuals born between September 1961 and September 1981.

**FIGURE 2:**
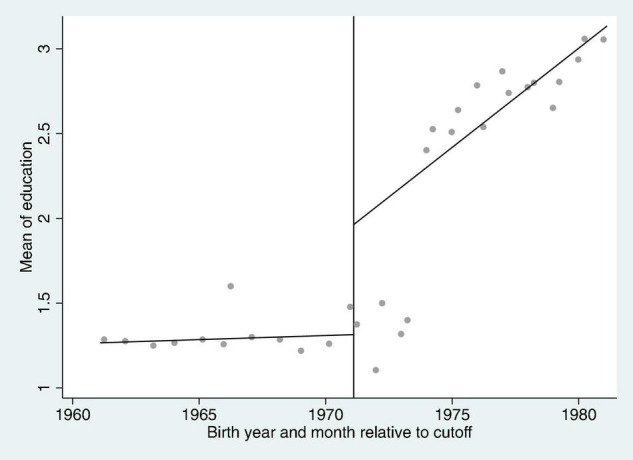
The education discontinuity of rural women after the implementation of the Compulsory Education Law.

Before implementing the RDD, the study first assessed the discontinuity caused by the Compulsory Education Law. The vertical line in Figure 2 is the dividing line for people affected by the 1986 Compulsory Education Law. The discontinuity in the figure is not clear in September 1971, given the fact that after the promulgation of the national law, the specific implementation documents at the local level were issued after one another. There was a certain time lag as each locality had to gradually advance and implement it according to the actual situation. So this study assumes that China’s compulsory education development has not reached the strict realization of 9-year compulsory education in those years. Therefore, this article employed a fuzzy design (Lee and Lemieux, 2010).

**FIGURE 3:**
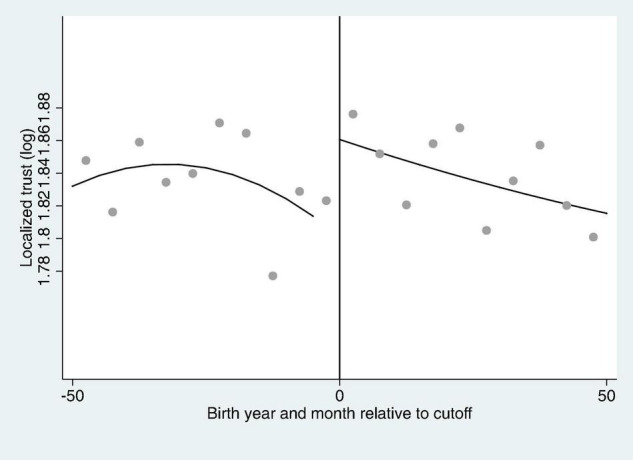
The localized trust (log) discontinuity of rural women after the implementation of the Compulsory Education Law.

**FIGURE 4:**
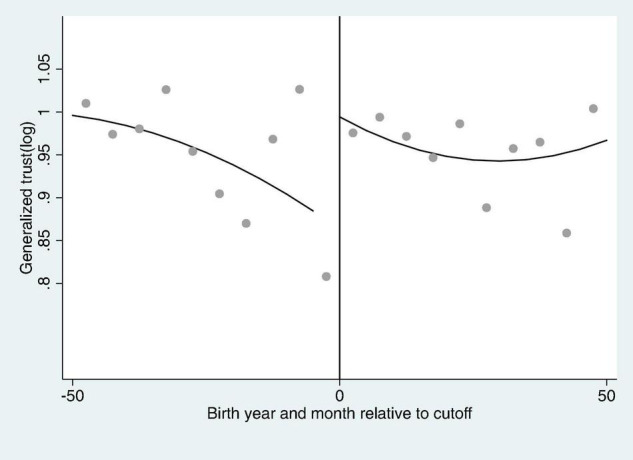
The generalized trust (log) discontinuity of rural women after the implementation of the Compulsory Education Law.

In Figure 3, the localized trust (log) discontinuity of rural women after the implementation of the Compulsory Education Law is shown. According to the figure, the localized trust of the people at the discontinuity is significantly higher as there is a localized trust discontinuity relative to the birth time of the “just-affected population” due to the implementation of the Compulsory Education Law. Moreover, Figure 4 also shows the generalized trust (log) discontinuity of rural women after the implementation of the Compulsory Education Law. Like localized trust, generalized trust also unveils significant rise at discontinuity point and imply that the causal relationship exists between education and trust.

**FIGURE 5:**
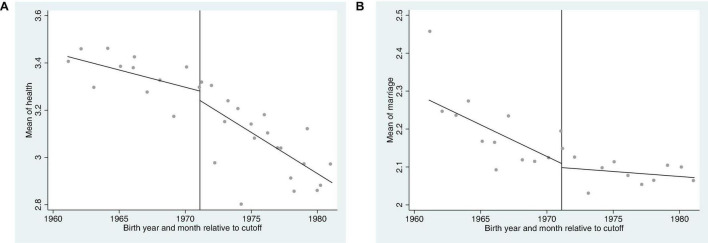
**(A,B)** The marriage and health discontinuity of rural women after the implementation of the Compulsory Education Law.

The effectiveness of RD also depends on the smoothness assumption. In addition to the years of education, other control variables such as marital status and health may also affect trust. So Figures 5A,B provide visual evidence for the marriage and health. It is apparent from Figure 5 and Table 5 that both variables have no obvious discontinuity near the discontinuity point, which meets the smoothness requirements.

## Discussion

The trust is of great significance to the economic and social development of a country. In the case of China, the trust of rural women in China has undergone tremendous changes along with the development of rural areas. It is argued that the trust of rural women has changed from localized to generalized trust, and it is believed that the major factor leading to this transformation is education. To explore this phenomenon empirically, the current study uses the survey data of rural women sourced from CFPS over the year 2018. Through the ordered probit model, the study reveals that education plays a significant role in influencing both generalized trust and localized trust. According to the empirical analysis, it is found that when rural Chinese women become more educated, their level of trust rises. The current study employed two types of trust, that is, generalized trust and localized trust. So the findings in the case of the generalized trust revealed that education plays a significant role in influencing rural women’s generalized trust and infers that rural women with higher education are more likely to trust strangers. It reflects the point that rural women with higher education are more likely to believe in the institutions and laws of modern society, so they are likely to believe in strangers more than their counter partners, that is, uneducated women or women with low educational levels. Moreover, the results are consistent with the findings of previous studies (Leigh, 2006; Borgonovi, 2012; Sønderskov and Dinesen, 2014), and differ significantly from Oskarsson et al. (2017)’s study. Sønderskov and Dinesen (2014) revealed that education is a key that can foster generalized trust. A higher reliance on the Internet and access to public resources in educated people will make them understand the benefits of the Internet and access to public resources. It is believed that the dissemination of information will make women understand stranger and more importantly trust each other. Moreover, when they will have more access to public resources locally, they will trust the government more and thus their generalized trust will be upsurged. Similar findings are found for localized trust and infer that rural women with higher education levels are more likely to trust their neighbors. The mechanism of education influencing localized trust revealed that mobility has a negative moderating effect between education and trust. Rural women with higher education are more likely to change their Hukou, however, they usually lack a sense of identity for themselves. These rural women do not consider themselves as a member of the cities, so they reduce localized trust. It is worth mentioning to explain the mindsponge mechanism to explain the change of trust of rural women. The mindsponge is a mechanism illustrating how a person can absorb new values and eject waning values conditionally based on contexts. Mindsponge is a broader, more inclusive model of the cognition shifting process (Vuong and Napier, 2015; Nguyen et al., 2021). According to mindsponge mechanism, we assume that every rural woman has a set of core cultural values and most of the relationships are based on blood, so in this case, they are believed to trust only relatives and acquaintances. Rural women use their core values as benchmarks to judge useful information or to make decisions with whom they are familiar with, and likely to accept their central circle. There is a zone out of the central circle protecting their mindset from external shocks, such as cultural novelty.

Moreover, the study additionally employed a heterogeneity test and found that the trust of rural women is different in space and time. The results of the effect of education on rural women’s trust are based on two groups, those who were born before 1980 and those who were born after 1980. It is believed that rural women’s trust shift from localized trust to generalized trust with the development of education. This phenomenon is more pronounced in younger people born after 1980 and in the more developed eastern and central regions of China. The possible reason is that rural women’s trust may change from localized trust to generalized trust more quickly in the economically developed central and eastern provinces, and the level of education may have a certain impact on the conversion process. Younger rural women living in these areas have been influenced more by the Internet. They use the Internet more frequently (such as online shopping and watching short videos) and are more likely to accept the new values. At the same time, they have less communication with those around them, so they are more likely to shift from localized trust to generalized trust. According to Fukuyama (1995), one of the characteristics of modern society is that people shift from localized trust to generalized trust. Since China’s reform and opening up, both material life and spiritual life have undergone major changes. Therefore, the changes in the general trust of the younger generation of rural women also reflect the changes in the Chinese society to a certain extent. On the contrary, the older women, who live in the backward areas, use the Internet rarely and have less interaction with anonymous people. The filtering mechanism may prevent them from entering into a new central circle and adopting new values, and they are more inclined to maintain the original attitude and trust in the people around them.

Furthermore, with the migration of a large number of rural laborers to cities and the popularization of the Internet in China, a large amount of new information and the new value disseminated to rural areas especially to rural women. In this vein, they are likely to compare the difference between the emerging and existing values and consider the costs and benefits of accepting or rejecting the emerging ones. For example, when online shopping appeared as a new thing, the filtering mechanism (in the mindsponge mechanism) is more likely to incorporate information that is compatible with rural women’s localized trust. The generalized trust is also believed to be widely accepted and acknowledged by them, because only in that case, transaction can be made. Thus, at some point, rural women have to change their trust from localized trust to generalized trust, especially in the changing world. For example, since the outbreak of the Coronavirus disease 2019 (COVID-19), COVID-19 vaccine is one of the most effective ways to effectively respond to the COVID-19 (Si et al., 2021b,c), and the effectiveness of vaccine distribution and injection heavily depends on public perception of the vaccines (Vuong et al., 2022). In this regard, trust (especially generalized trust) plays a key role regarding how to convince rural residents, especially rural women who are relatively unaware about the role of vaccines. So trust plays an important role in all spheres of life. Finally, the RDD also confirmed the causality between education and trust.

## Conclusion

Over the past 40 years, tremendous progress has been made in rural China in the context of education (Yue et al., 2018), and education is regarded as a major factor leading to trigger the trust of rural women in China. The development of rural areas mainly depends on the modernization of people and in this vein, trust plays crucial role in human modernization. It is believed that rural women’s trust has changed from that based mainly on blood relationships to society, and community. In the current study, it is found that education has a significant positive effect on rural women’s trust in China. And the mechanism also revealed that reliance on Internet information, access to public resources, and income have a mediating effect between education and generalized trust, and mobility has a moderating effect on localized trust. Besides, the heterogeneity test results further unveil that the younger rural women who live in areas with more developed economies have more generalized trust. The RDD also found the causality between education and trust. The overall findings confirmed that the higher the level of education boosts the trust of rural women in China. Based on the findings, the study proposes that measures should be taken to help rural women to receive more education, especially 9-years compulsory education, as according to the study of Liu and Rozelle (2020), the dropout rate from junior high school is still high (14%) in 2011. Government is required to invest in rural education strategies and encourage rural women to acquire education. Consequently, it will not only lead to boosting the rural women’s trust but also maintain cooperation among people for the sustainable development of the country.

## Limitations of the Study

This study is not without limitations. First, a few studies consider trust as a dynamic process (Paliszkiewicz, 2011), but this article uses cross-sectional data for justifying assumptions on a dynamic mechanism for the data limitations. Second, the transformation of rural women’s trust may be the effect of the superposition of traditional culture and Internet culture. In future research, quantitative calculation of the size of this superimposed effect can be considered (Vuong et al., 2018). In a changing society, women in developing areas are facing multiple cultural shocks and reshaping of value systems, so not just trust, but many facets of change are worth studying (Aziz et al., 2021b). Third, due to the limitation of data, the selection of some proxy variables may not be accurate enough, which can be optimized in future research. Moreover, our study only discussed the rural women’s trust through the individual level, but the effect of institution and culture on trust cannot be ignored in China. Only a few studies explored this phenomenon (Rothstein and Stolle, 2008; Sønderskov and Dinesen, 2014), so future research may focus on discussing the relationship between institutions and trust, and elucidate how institution affects trust. Moreover, in the pandemic era, trust (especially generalized trust) plays a key role to convince rural residents, especially rural women to vaccinate themselves. So in this regard, trust also set avenues for future researchers to discuss the relationship between trust and COVID-19 vaccines generally and especially in the case of rural women.

## Data Availability Statement

The original contributions presented in the study are included in the article/supplementary material, further inquiries can be directed to the corresponding author/s.

## Author Contributions

SX wrote the main article. YZ and NA revised and reviewed the article. JH supervised the article. All authors contributed to the article and approved the submitted version.

## Conflict of Interest

The authors declare that the research was conducted in the absence of any commercial or financial relationships that could be construed as a potential conflict of interest.

## Publisher’s Note

All claims expressed in this article are solely those of the authors and do not necessarily represent those of their affiliated organizations, or those of the publisher, the editors and the reviewers. Any product that may be evaluated in this article, or claim that may be made by its manufacturer, is not guaranteed or endorsed by the publisher.
